# Wavelet principal component analysis of fetal movement counting data preceding hospital examinations due to decreased fetal movement: a prospective cohort study

**DOI:** 10.1186/1471-2393-13-172

**Published:** 2013-09-05

**Authors:** Brita Askeland Winje, Jo Røislien, Eli Saastad, Jorid Eide, Christopher Finne Riley, Babill Stray-Pedersen, J Frederik Frøen

**Affiliations:** 1 Division of Epidemiology, Norwegian Institute of Public Health, PO Box 4404, Nydalen, 0403, Oslo, Norway; 2 Department of Biostatistics, Institute of Basic Medical Sciences, University of Oslo, Oslo, Norway; 3 Department of Obstetrics and Gynecology, Østfold Hospital Trust, Fredrikstad, Norway; 4 Institute of Clinical Medicine, University of Oslo, Oslo, Norway; 5 Women and Children’s Division, Oslo University Hospital Rikshospitalet, Oslo, Norway

**Keywords:** Decreased fetal movement, Fetal movement counting, Fetal movement chart, Kick chart, Kick counting, Fetal monitoring, Fetal compromise, Wavelet principal component analysis

## Abstract

**Background:**

Fetal movement (FM) counting is a simple and widely used method of assessing fetal well-being. However, little is known about what women perceive as decreased fetal movement (DFM) and how maternally perceived DFM is reflected in FM charts.

**Methods:**

We analyzed FM counting data from 148 DFM events occurring in 137 pregnancies. The women counted FM daily from pregnancy week 24 until birth using a modified count-to-ten procedure. Common temporal patterns for the two weeks preceding hospital examination due to DFM were extracted from the FM charts using wavelet principal component analysis; a statistical methodology particularly developed for modeling temporal data with sudden changes, i.e. spikes that are frequently found in FM data. The association of the extracted temporal patterns with fetal complications was assessed by including the individuals’ scores on the wavelet principal components as explanatory variables in multivariable logistic regression analyses for two outcome measures: (i) complications identified during DFM-related consultations (*n* = 148) and (ii) fetal compromise at the time of consultation (including relevant information about birth outcome and placental pathology). The latter outcome variable was restricted to the DFM events occurring within 21 days before birth (*n* = 76).

**Results:**

Analyzing the 148 and 76 DFM events, the first three main temporal FM counting patterns explained 87.2% and 87.4%, respectively, of all temporal variation in the FM charts. These three temporal patterns represented overall counting times, sudden spikes around the time of DFM events, and an inverted U-shaped pattern, explaining 75.3%, 8.6%, and 3.3% and 72.5%, 9.6%, and 5.3% of variation in the total cohort and subsample, respectively. Neither of the temporal patterns was significantly associated with the two outcome measures.

**Conclusions:**

Acknowledging that sudden, large changes in fetal activity may be underreported in FM charts, our study showed that the temporal FM counting patterns in the two weeks preceding DFM-related consultation contributed little to identify clinically important changes in perceived FM. It thus provides insufficient information for giving detailed advice to women on when to contact health care providers. The importance of qualitative features of maternally perceived DFM should be further explored.

## Background

Most women are aware of fetal movement (FM) and notice changes in its intensity and frequency [1]. Decreased fetal movement (DFM) causes concern [1,2] and often leads to unscheduled antenatal consultation [3,4], which consumes significant health care resources and remains a challenge in obstetric care. Although the majority of pregnancies with perceived DFM continue without complication [5], maternal concern should be taken seriously because DFM has been linked to a wide range of adverse birth outcomes, including fetal growth restriction (FGR) and death [6-10].

FM counting, in which the mother systematically records FM, has been suggested as a tool to improve maternal self-screening for DFM [8,11,12]. The daily routine of FM counting may improve a woman’s ability to identify alarming changes in FM in a timely manner, enabling appropriate intervention if the fetus is at risk. Although this method is simple and feasible, its use remains controversial, mainly because no limit for clinically important DFM has been adequately defined [11,12]. Moreover, no counting method or DFM limit has been proven to be superior to maternal perception of DFM [11,12].

Although FM must be understood through the mother, few studies have examined the association between perceived DFM and actual FM counts. The analysis of FM counting time series is complex. Due to methodological shortcomings, studies to date have mainly focused on fixed DFM-limits and their ability to identify risk, although substantial individual variation in fetal activity cautions against this approach [12-14]. Also, fixed DFM-limits cannot capture individual temporal patterns in counting series, such as emerging trends, shifts and changes in variability, which could provide important information about fetal well-being [15]. To explore clinically important changes in temporal FM patterns, a better understanding of what women perceive as DFM and how this is related to adverse outcomes is needed.

This study reports data from the prospective *Count with Me* study initiated by the Norwegian Institute of Public Health in 2009 as part of the international Fetal Movement Intervention Assessment (*FEMINA*) research collaboration [2-4]. FEMINA covers various aspects of fetal movement monitoring for improving perinatal outcomes. The focus of the *Count with Me* study is the analyses of FM counting charts to explore whether they contain clinically important information that may improve maternal self-screening.

In order to unveil common temporal patterns across individual FM charts prior to perceived DFM, we applied wavelet principal component analysis [16]. Wavelets are an important tool in signal analysis and have previously been used in medical research fields such as electromyography [17] and neural behavior [18,19]. It allows for localized feature extraction from a time-varying signal, including not only various long-term trends but also sudden temporal changes, i.e. spikes that are frequently found in FM data. The PCA extracted a set of common components that captured the main variation in the data across the individual FM charts.

Using this novel statistical methodology, specifically developed for this study, we aimed to explore common temporal patterns in FM charts in the two weeks preceding hospital examination due to DFM, and whether these patterns were associated with fetal complications and placental histopathology.

## Methods

### Setting and population

The study was conducted in collaboration with the Østfold Hospital Trust, a hospital serving the total population of Østfold County handling approximately 3000 births annually. Between July 2009 and July 2011, all women attending Østfold Hospital Trust for routine ultrasound screening in pregnancy weeks 17–19 who had sufficient Norwegian literacy to understand the FM counting protocol were invited to participate in the *Count with Me* study. A total of 2468 women (41% of eligible participants) were enrolled in the study, and the 1445 (59%) women who submitted FM charts were included in the study group. This paper reports on FM counting data from a subset of 207 women (14% of the study group) who were examined due to perceived DFM after pregnancy week 24.

**Figure 1:**
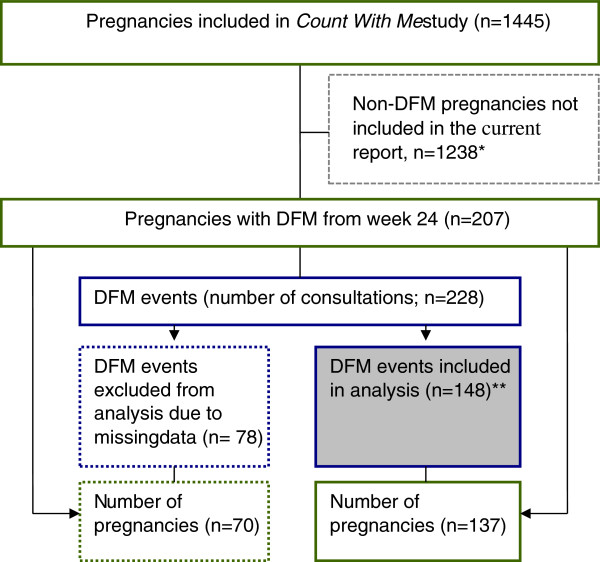
**Flow chart of data selection.** DFM, decreased fetal movement. *Two of 150 events (second consultations) in this group were excluded due to insufficient data.

Our unit of analysis was FM counting patterns in the two weeks preceding a DFM event, defined as a hospital visit for the evaluation of perceived DFM causing maternal concern. In total, there were 228 DFM events (Figure 1). For the purpose of studying FM counting patterns in the period preceding the DFM event, we delimited the subset to DFM events where women had sufficient counting observations recorded. We defined this as having observation recorded *at the day prior to* or *on the day of* the consultation and at least one additional counting observation in the two weeks preceding DFM. In total 148 DFM events from 137 pregnancies met the compliance criteria and were included in the analysis. Complete counting observations from the two weeks preceding DFM-related consultations were available for 61/148 (41%) DFM events, and one to nine observations from this period were missing for the remaining events. Observations from the day of consultation were missing for 30 (20%) events, which is higher than the median of 13 (range, 10–23) missing records for the remaining days.

The proportion of consultations where fetal pathology was identified at the DFM examination was similar between DFM events included in the analysis and those excluded due to low compliance, 15% in both groups. Maternal characteristics and obstetric indicators are presented in Table 1.

**Table 1:** Characteristics of pregnancies with and without maternal concern about decreased fetal movement (DFM)

**Characteristics, n (%)**	**DFM pregnancies included in analyses, n = 137**	**DFM pregnancies with consultations within last 21 days prior to birth n=76**	DFM pregnancies excluded from analyses, n= 70*	Non-DFM pregnancies, n=1238**
	**n (%)**	**n (%)**	**n (%)**	**n (%)**
**PRE PREGNANCY**				
**Maternal characteristics**				
Maternal age ≥ 35 years	26 (19)	19 (25)	11 (16)	200 (16)
Primiparous	74 (54)	35 (46)	44 (63)	645 (52)
Maternal obesity (body mass index ≥ 30 kg/m^2^)	21 (15)	11 (15)	14 (20)	172 (14)
Daily/occasionally smoking 1^st^ trimester	7 (5)	4 (5)	14 (20)	114 (9)
**Obstetric/general health risk factors**^*a*^	26 (19)	18 (24)	4 (6)	110 (9)
**DELIVERY AND BIRTH OUTCOME**				
**Delivery complications**				
Intrapartum interventions due to non-reassuring fetal state^*b*^	7 (5)	3 (4)	7 (10)	157 (13)
Emergency cesarean section^*c*^	16 (12)	7 (9)	7 (10)	139 (11)
**Birth outcomes**				
Healthy^*d*^	69 (50)	40 (53)	36 (51)	611 (50)
Neonatal complications^*e*^	28 (20)	18 (24)	14 (20)	250 (20)
Intrauterine fetal death	1 (1)	1 (1)	-	2 (0.2)
Small for gestational age^*f*^	13 (10)	9 (12)	11 (16)	127 (10)
Fetal growth restriction^*g*^	3 (2)	3 (4)	4 (6)	31 (3)
Apgar <7_5minutes_	2 (2)	2 (3)	-	20 (2)
Preterm birth (week 24^0^ – 36^6^)	12 (9)	9 (12)	1 (1)	60 (5)

Data are reported as n (%). Numbers from column one (n=137), column three (n=70) and column four (n=1238) form the total *Count with Me* study cohort (n=1445).

*There were in total 207 DFM pregnancies; DFM events from 70 pregnancies were excluded due to low counting compliance.

** In total 1238 non-DFM pregnancies were part of the *Count with Me* study, but are not included in the current report.

^a^Obstetric risk factors: Previous pregnancy with fetal growth restriction, stillbirth > 21 weeks, fetal malformations, severe preeclampsia, preterm delivery, and/or > 3 spontaneous abortions. General maternal health risk factors: known type I or II diabetes, chronic renal, hypertensive or coronary disease, inflammatory and collagen disease, epilepsy, hypothyreosis or coagulopathy. Data were collected from medical records.

^b^Asphyxia or protracted delivery with pathological cardiotocography finding.

^c^Intervention decided upon within eight hours before delivery, including acute and emergency cases.

^d^No pathology identified at DFM-related consultation, uncomplicated pregnancy ending in spontaneous vaginal term delivery of a healthy infant with birth weight > 10^th^ percentile (adjusted for gestational age and sex), Apgar score >7_5min,_ no neonatal complication or transfer to neonatal care unit and normal placental examination findings.

^e^Small for gestational age, infections, Apgar score <7_5min,_, malformations or transfer to neonatal care unit for conditions relevant to growth restriction or fetal distress (respiratory syndrome or cerebral irritation).

^f^Birth weight < 10^th^ percentile, adjusted for gestational age and fetal sex.

^g^Birth weight < 2.5^th^ percentile, adjusted for gestational age and fetal sex.

### Ethical approval

This study was approved by the Regional Committee for Medical Research Ethics (S-08694d, 2008/18353, 06.26.2009). All participants provided written informed consent.

### Instruments and measures

**Figure 2:**
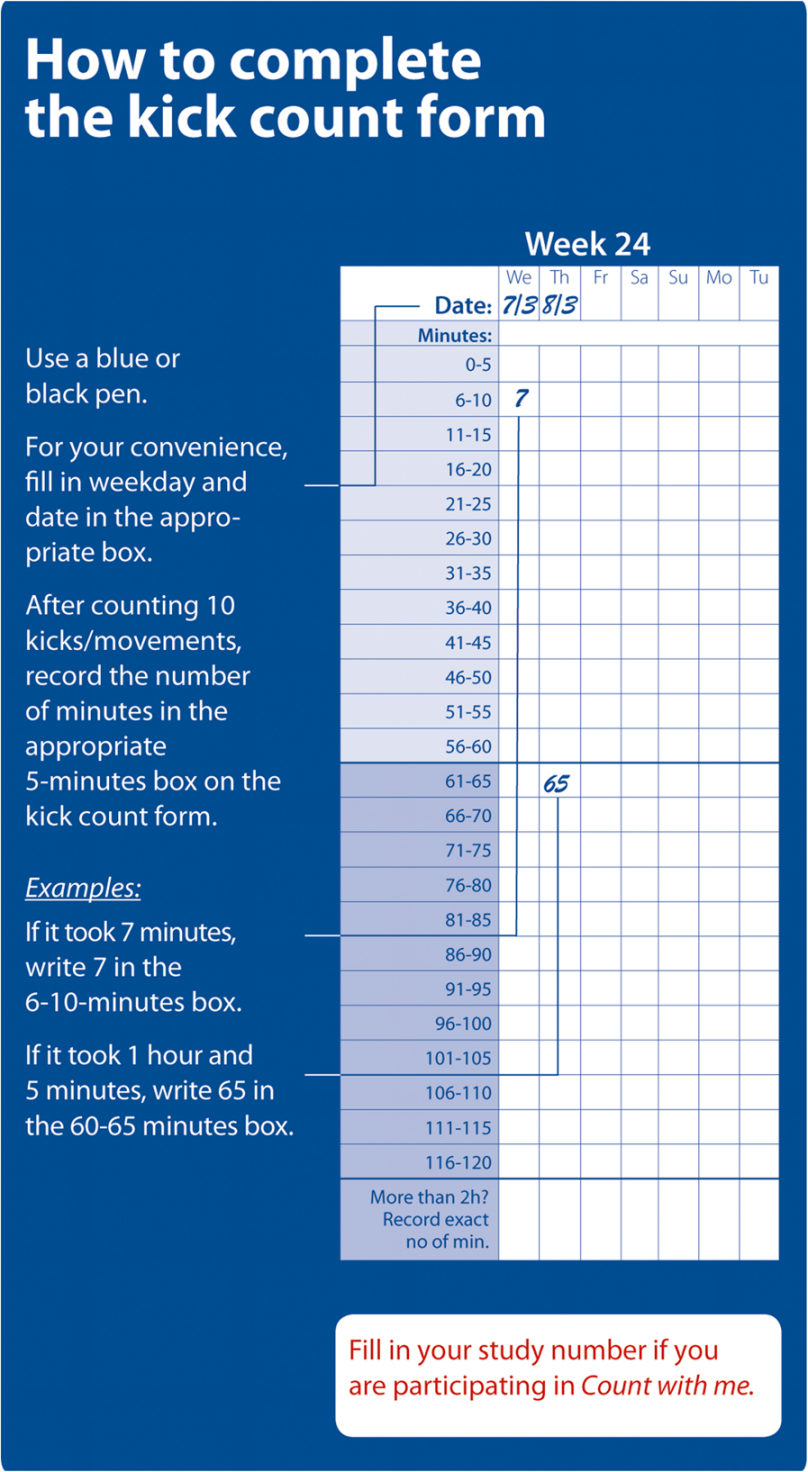
**Instructions to women on how to record fetal movement counts in the charts.** This figure is included as part of the fetal movement chart.

Demographic and obstetric information for each participant was obtained from antenatal pregnancy charts and hospital records. The details and rationale for the FM counting method used in this study (FEMINA protocol) have been presented in detail previously [20]. A designated research midwife informed participants about FM and instructed them in the use and interpretation of the FM chart (Additional file 1). Each woman was instructed to count FM daily from pregnancy week 24 until delivery using the count-to-ten procedure. She was encouraged to count within the same two hour period every day at a time when she knew her baby was usually active. The mother initiated counting when she perceived the first movement, and then recorded the time needed to count the additional nine movements (in minutes) on the FM chart (Figure 2). All movements counted as kicks, simultaneous kicks and rolling movements counted as a single kick, and hiccups were disregarded. Women were advised to be attentive to significant and sustained reductions in normal fetal activity, which took priority over any formal DFM limit. If women were worried about their baby, regardless of reason, they should seek advice and help from their doctor or midwife. If they were concerned because their baby was less active as weeks went by, they should bring their kick count form to their next pregnancy check-up. They were instructed to contact their maternity unit directly if the baby did not kick one day (never wait until the next day) or if the baby kicked less during day/days and they perceived decreased fetal movement. They were informed that a healthy baby rarely kicks fewer than 10 times within a 2-hour period when the baby is normally active.

DFM-related examinations included cardiotocography and biophysical profiling in the majority of cases and Doppler ultrasound when indicated. Clinical management followed the hospital’s routine clinical care protocol. Maternal and fetal complications were classified according to Norwegian guidelines for antenatal care [21], which are largely consistent with the Royal College of Obstetricians and Gynaecologists (RCOG) Green Top Guidelines [22].

Our main outcome measures were (i) the identification of fetal complications at DFM-related consultations (yes/no) and (ii) the presence of fetal compromise at the time of DFM-related consultation (yes/no). The former measure reflected only the outcome of the examination, whereas the latter included information from medical files, birth outcome data, and placental pathology findings. Whether adverse birth outcome or placental pathology in retrospect was assumed relevant to the DFM consultation was based on the underlying pathology and the time between the DFM consultation and delivery, and was assessed independent of the FM information. It was, however, restricted to DFM consultations occurring within 21 days before birth.

Small for gestational age (SGA) was defined as birth weight < 10 percentile and FGR as birth weight < 2.5 percentile, adjusted for gestational age and sex [23-25]. Fetal complications identified at the DFM consultation included fetal death, fetal distress (non-reassuring cardiotocographic finding or pathological blood flow in umbilical artery), poly- or oligohydramnios (as reported in clinical files), fetal weight estimate <−10% by ultrasound measurement or fetal malformations. The composite outcome measure of fetal compromise at time of DFM consultation included: (i) fetal complications as listed above, (ii) intrapartum interventions due to non-reassuring fetal state (asphyxia or protracted delivery with pathological cardiotocographic finding) or emergency cesarean section, (iii) neonatal complications including death, SGA, FGR, Apgar <7_5min_, or other relevant complications, or (iv) placental pathology.

We used a strict definition of healthy pregnancy including normal outcome of a DFM-related examination, followed by spontaneous vaginal term delivery of a healthy infant with birth weight > 10^th^ percentile (adjusted for gestational age and sex, [23-25]), Apgar score ≥ 7_5min_, absence of neonatal complication or transfer to the neonatal care unit, and normal findings of placental examination.

### Placental pathology

A placenta sub-study was an integral part of the *Count with Me* study. The sub-study had two entry points: (i) women preselected to a population cohort at the time of enrollment to the fetal movement counting study, and (ii) women examined in hospital for DFM during pregnancy. The management of placenta samples has been described in detail previously [26], and only a condensed version is presented here. Placentas were examined according to standardized macro- and microscopic protocols. For focal lesions, the estimated percentage of total placental volume, location (central or peripheral), and arbitrarily defined timing (acute, hemorrhagic changes within <48 hours; subacute, hemorrhagic and fibrous changes within 2–20 days; longstanding, fibrous changes within ≥21 days) were recorded. Infarctions with clinical impact were defined as those occupying ≥5% central or ≥10% peripheral placental volume. Morphological findings were classified using a new Norwegian system for reporting placental pathology [27] and timed accordingly. For this study, only placental pathologies with moderate to significant clinical impact were included as pathological findings in the analyses [26].

Placentas from 62% of DFM pregnancies in the *Count with Me* study were eventually collected. There were no significant differences in mean infant birth weight, mean gestational age at birth, neonatal complications, SGA or preterm birth between DFM pregnancies with and without placentas collected [26]. The DFM placentas missed in the study were most likely random. In the present study, placentas were available from 55 of the pregnancies included (61%).

### Statistical analyses

Descriptive measures of continuous variables are presented as means and standard deviations for symmetrical data, and as medians and ranges for skewed data. Descriptive measures of categorical variables are presented as frequencies and percentages.

To identify similarities in temporal patterns across individual FM charts, we used wavelet principal component analysis (PCA) [16]. This novel comprehensive statistical procedure, developed specifically for this study, handles missing data by multiple imputation [28,29]; individual FM charts are modeled using wavelets [30,31], enabling the extraction of localized features from time-varying signals and of common temporal patterns, such as shifts, trends, and spikes, by PCA [32]. PCA is a multivariate technique that reveals the internal structure of data in a way that best explains variance in the data. The wavelet PCA also generates a set of scores for each woman characterizing the extent to which each main temporal pattern is represented in her individual FM chart. These scores were then included as continuous explanatory variables in standard multiple logistic regression analyses to explore their associations with adverse outcomes.

We conducted two separate wavelet PCAs. The primary analysis included the full cohort of 148 DFM events, and the secondary analysis included a subsample of 76 DFM events occurring within 21 days before birth. Scores from each of the three first temporal principal components were included as continuous explanatory variables in two separate logistic regression analyses, with (i) outcome of DFM-related consultation, and (ii) fetal health at the time of DFM consultation serving as dependent variables, respectively. Gestational age on the day of consultation differed considerably among the DFM events. As a determinant for FM counting patterns, gestational age was included as a continuous explanatory variable in the analyses. In the former analysis, we also fitted a fixed mixed model [33] to adjust for multiple DFM-related consultations within pregnancies.

Wavelet principal components other than the first three were excluded from regression analyses because they explained little of the total variation in the data and were increasingly difficult to interpret clinically. We also calculated the number of DFM events with fewer than 10 FM in one and two hours respectively. All statistical analyses were performed using R 2.12 software [34]. *P*-values < 0.05 were considered to be statistically significant.

## Results

Maternal characteristics and obstetric indicators are presented in Table 1. Complications were identified in 22/148 (15%) of the DFM-related consultations (Table 2). In 15 (68%) cases, the complication was not identified prior to the consultation. Taking birth outcome and placental pathology into account, fetal compromise relevant to the DFM consultation was identified in 27/76 (36%) cases. Placental infarction (*n* = 6) and villitis (*n* = 1) helped to potentially explain seven otherwise unexplained DFM events.

**Table 2:** Fetal complications identified during consultations due to decreased fetal movement (DFM)

**ALL DFM-RELATED CONSULTATIONS** (n=148)		
Outcome of hospital examination due to DFM	PATHOLOGY IDENTIFIED AT DFM-RELATED CONSULTATION	22 (15%)
	Intrauterine fetal death	1
	Fetal distress^*a*^	4
	Polyhydramnios^*b*^	1
	Oligohydramnios^*b*^	2
	Fetal weight estimate <-10% by ultrasound measurement	14
	Fetal malformation	1
**DFM-RELATED CONSULTATIONS 21 DAYS BEFORE BIRTH** (n=76)		
Proxy for fetal health at time of consultation, based on examination outcome, birth outcome and placental pathology assumed relevant to the consultation	ASSUMED FETAL COMPROMISE AT TIME OF CONSULTATION	27 (36%)
	**Pathology identified at DFM-related consultation**	19
	**Delivery complications**	
	Intrapartum intervention due to non-reassuring fetal state^*c*^	0
	Emergency cesarean section^*d*^	3
	**Birth outcome**	
	Neonatal complications^*e*^	11
	Intrauterine fetal death	1
	Small for gestational age^*f*^	7
	Fetal growth restriction^*g*^	3
	Apgar <7_5minutes_	2
	Other	3
	**Placental pathology, total** [*n*=48 (63%)]	13
	Infections^*h*^	1
	Maternal placental circulatory disorder^*i*^	10
	Other	2

^a^Non-reassuring cardiotocography finding or pathological blood flow in umbilical artery (as defined by clinician).

^b^As reported by clinicians in medical records.

^c^Asphyxia or protracted delivery with pathological cardiotocography finding.

^d^Intervention decided upon within eight hours before delivery, including acute and emergency cases.

^e^Small for gestational age, infections, Apgar score <7_5min,_ malformations or transfer to neonatal care unit for conditions relevant to fetal growth restriction or fetal distress (respiratory syndrome or cerebral irritation).

^f^Birth weight < 10^th^ percentile, adjusted for gestational age and fetal sex.

^g^Birth weight < 2.5^th^ percentile, adjusted for gestational age and fetal sex.

^h^Chorioamnionitis or villitis.

^i^Infarctions/lesions, hemorrhages, abruptions and ischemic changes.

**Figure 3:**
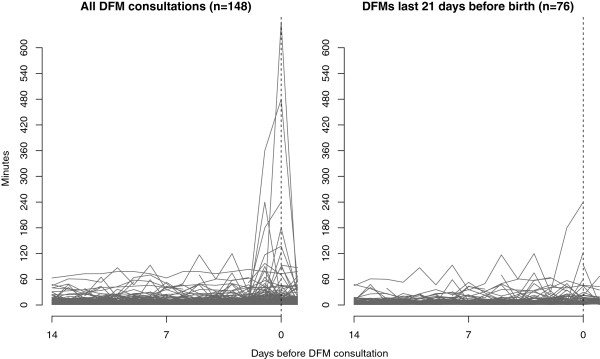
Fetal movement counts two weeks preceding consultations due to decreased fetal movement (DFM).

FM count data from the two weeks preceding DFM events are shown in Figure 3. These data show a large degree of individual variation. Despite missing observations on the day of DFM consultation in 20% of cases, sudden temporal changes represented by spikes, was visible around the day of DFM-related consultation for several women. In many cases, counting times decreased again following clinical examination.

**Figure 4:**
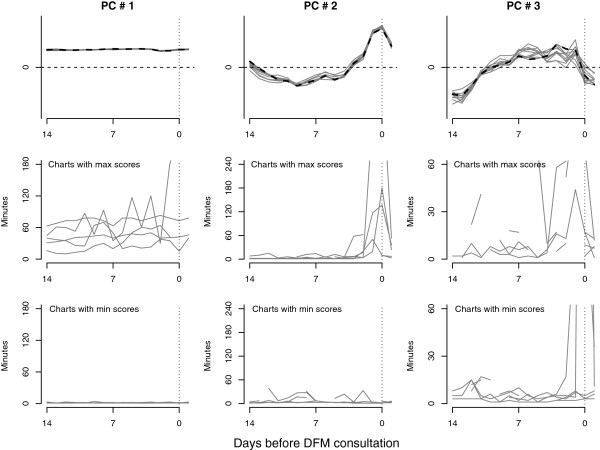
**Wavelet principal component curves for fetal movement preceding consultations due to decreased fetal movement.** Curves are shown for the first three principal components (PCs), drawn from 148 fetal movement counting time series from the two weeks preceding DFM-related consultation, together with the five highest (max) and lowest (min) scores for each PC. DFM, decreased fetal movement.

Running wavelet PCA for all 148 FM counting series, the first three temporal principal component curves explained 75.3%, 8.6%, and 3.3% (total, 87.2%) of the total variation among FM counting charts, respectively. These three temporal components are shown in Figure 4, together with the FM charts yielding the five highest and five lowest scores, respectively, for each temporal principal component. Similar temporal components were identified for the subset of 76 consultations occurring within 21 days before birth (data not shown). For this subsample, the first three temporal principal components explained 72.5%, 9.6%, and 5.3% (total, 87.4%) of the total variation among FM charts.

In both wavelet PCAs, the first and by far most dominant temporal component mainly represented the levels of FM curves relative to the overall mean. A high score on this component implied longer than average counting times, and a low (very negative) score implied shorter than average counting times. The second temporal component captured a spike in the data around the time of DFM-related consultation. A high score on this component implied that suddenly longer counting time surrounded the DFM-related consultation, whereas a low (very negative) score implied no such spike. Because most women counted FM in the evening hours, the cluster of spikes occurred around the time of DFM-related consultation, not specifically on the consultation day. The third temporal component described an inverted U-shaped pattern, implying that a DFM event followed a period of higher than average counting times in the middle of the preceding two-week period. Low (very negative) scores on this component implied shorter counting times during this period.

Multiple logistic regression analyses revealed that no temporal principal component was significantly associated with pathology identified during DFM-related consultation in the full cohort (*n* = 148) or fetal compromise at the time of consultation in the subsample of 76 consultations occurring within 21 days before birth (Table 3).

**Table 3:** Multivariable logistic regression models for associations between fetal movement patterns and fetal health

**CONSULTATIONS DUE TO DECREASED FETAL MOVEMENT,***(n =148)***OUTCOME: pathology identified at DFM consultation**^***a***^						
	**Simple regression**			**Multivariable regression**		
**Continuous explanatory variables**	**Estimate**	***p***	**Estimate**	**SE**	***p***	**95% CI**
Wavelet PC scores						
PC1: general level	0.096	0.163	0.053	0.038	0.208	-0.02 to 0.13
PC2: spike around day of consultation	-0.159	0.164	-0.166	0.128	0.237	-0.41 to 0.08
PC3: inverted U-shape	0.251	0.149	0.294	0.179	0.143	-0.06 to 0.65
Gestational age on day of consultation (days)	0.015	0.106	0.019	0.010	0.093	-0.00 to 0.04
**CONSULTATIONS DUE TO DECREASED FETAL MOVEMENT WITHIN 21 DAYS BEFORE BIRTH,** (*n*=76) **OUTCOME: Fetal compromise at DFM consultation**^***b***^						
Wavelet PC scores						
PC1_21: general level	0.066	0.104	0.062	0.043	0.144	-0.02 to 0.15
PC2_21: spike around day of consultation	- 0.070	0.566	-0.103	0.138	0.455	-0.37 to 0.17
PC3_21: inverted U-shape	0.245	0.177	0.308	0.194	0.113	-0.07 to 0.69
Gestational age on day of consultation (days)	- 0.017	0.260	-0.014	0.016	0.361	-0.05 to 0.02

SE, standard error; CI, confidence interval, PC, principal component; DFM, decreased fetal movement.

^a^Outcome (binary dependent) variable = pathology identified at consultation (22/148 events). Analysis included fitting a mixed-effects model to account for multiple consultations within pregnancies. Women in the sample had one (n=127), two (n=9), or three (n=1) DFM-related consultations. Mean gestational age at time of consultation was 250 days (range, 184-292 days; standard deviation, 31 days).

^b^Outcome (binary dependent) variable = fetal compromise at time of consultation (27/76 events). No women attended more than one consultation within 21 days before birth. Mean gestational age at time of consultation was 271 days (range, 213-292; standard deviation, 16 days).

In seven (5%) cases, counting observations corresponded to the DFM limit of “fewer than 10 movements within 2 hours” by Moore and Piacquadio [35]. Fewer than 10 movements within 1 hour were recorded in 21 (14%) cases.

## Discussion

To the best of our knowledge, this study is the first to report prospective FM counting data from the weeks preceding hospital examination due to DFM. Using the novel statistical approach of wavelet PCA, we found that most temporal variation in FM counting charts was related to differences in the overall temporal mean. Spikes around the time of DFM-related consultation also explained a fair amount of the observed variation, but they were unrelated to the adverse outcomes under study. Our results suggest that maternal concern about DFM arises due to factors other than sudden extreme changes in FM.

Previous total-population DFM studies have typically been retrospective, in which mothers seeking medical services due to DFM-related concern were recruited without any preceding FM counting [4,7,9,36,37]; or prospective FM counting studies, in which mothers were provided with fixed DFM limits and instructions on when to seek medical attention [35,38-42]. However, such limits have performed poorly when used for screening [11,12,20]. Not only are they scientifically questionable [12], but women also fail to comply with them; only 46% [39] and 63% [14] of women consulted antenatal care when alarms occurred and 6% [14] sought consultation in the absence of an alarm. Thus, other features of the observed temporal FM counting patterns may play a role in women’s perceptions of DFM. Although recognized by several authors [12-14], temporal patterns have not been adequately addressed, due to methodological constraints [15].

When modeling temporal phenomena, there is a balance between removing random variation, i.e. noise, while still retaining signals with potential clinical significance. In our setting, the smoothing of natural day-to-day variability that presumably means nothing had to be weighed against the need to maintain sufficient sensitivity for early signs of fetal compromise, particularly given our focus on DFM events and the overall short mean counting time in the sample. The wavelet approach proved to be useful in modeling temporal FM counting patterns (e.g., general levels and spikes) in contrast to previously applied temporal methods, such as functional data analysis, which tend to smooth out potentially important spikes [15].

PCA is often used to decompose variation in data. Extracting a set of common components that capture the main variation in the data makes the analysis easier to manage and interpret. Women were advised to be attentive to significant and sustained reductions in normal fetal activity for her baby. Still, the general levels of FM curves relative to the overall mean explained by far most variation in the FM charts, implying little change over time. Maternal concern for fetal well-being may be related to factors not directly linked to fetal activity. Women included in this analysis were more likely to have pre-existing obstetric or general health risk factors than non-DFM pregnancies, which could have made them more alert to changes in FM, resulting in the reporting of clinically insignificant FM changes. In addition, DFM events may have been masked in the FM charts because mothers may have experienced DFM, been examined, and been discharged with a normal outcome within a 24–hour window. We observed that for women with high counting times on the day on or before a DFM related consultation, counting time was back to her general level the day following the consultation. This could represent natural variation in individual counting times. Also, women could have been reassured by the outcome of the clinical examination or better informed about how to count FM during the hospital visit.

Most importantly, however, quantitative FM counting may not adequately reflect changes in the qualitative properties of FM, such as movement strength, speed, and complexity. Qualitatively abnormal general movements are frequent in compromised fetuses and correlate with hypertensive disorders and oligohydramnios [43]. Women’s premonitions prior to *in utero* fetal death included the suspicion that “something had changed” and the feeling that the “baby somehow floated around” [44]. These findings indicate that the quality of FM may be an important factor in identifying fetal compromise.

The observed spikes in FM chart data may explain DFM-related concern among affected women, but they were unrelated to adverse outcomes in our study. However, our findings do not imply that sudden changes in FM should be ignored as alarming signs. First, with few exceptions, the spikes represented modest changes (in terms of minutes) and did not necessarily represent alarming deviations from normal activity. This interpretation is confirmed by the small numbers of women with counting times exceeding one and two hours. Second, and more importantly, counting data were frequently missing on the day of DFM-related consultation, a natural response under the circumstances. As a result, acute and clinically important alarms may have been underreported. These findings have two important implications. First, women’s concerns will always remain vital. Second, FM patterns can be considered reassuring only when FM counting charts are complete. Whether the positive effects reported in FM counting studies [45,46] are due to increased FM vigilance through the daily routine of counting or to information contained in FM charts remains unresolved. Previous studies have been unable to disentangle these effects [11,46].

A substantial part of the FM charts had missing counting observations. Low compliance remains a challenge in FM counting studies [14,15,20,47]. Whether women omitted to count FM or whether they only omitted to record their counting observation in the chart is unknown. While both may affect the validity of our analysis, maternal awareness is less affected by the latter. We found that placental infarctions helped to explain otherwise-unexplained DFM events. This measure was probably underestimated because placentas were available only from 61% of participants.

In our study, 14% of women were examined in the third trimester of pregnancy due to DFM-related concern; this percentage was higher than the 4–13% range reported in previous studies [5,46]. However, most previous reports included only consultations for the primary complaint of DFM occurring after pregnancy week 28; these criteria apply to 11% of pregnancies in our study.

Women who seek health care due to concern about DFM are at risk of pregnancy complications [9,12,37,45,46]. Within this risk group, FM counting patterns in the two weeks preceding DFM events did not help to identify pregnancies at highest risk. Our future analyses of temporal FM counting patterns will explore whether patterns present in a total population may perform better than women alone in defining clinically important DFM. Self-screening by women continues. Because DFM concerns so many women, even a small improvement in the interpretation of FM may well have substantial impact on antenatal care and perinatal outcomes.

## Conclusions

The temporal FM counting patterns identified in data from the two weeks preceding DFM-related consultations contributed little to inform on clinically important changes in FM in this subgroup of risk pregnancies. Our study thus provides insufficient information for giving detailed advice to women about when to contact health care providers for DFM concerns. The importance of qualitative properties of maternally perceived DFM should be further explored.

## Abbreviations

DFM: Decreased fetal movement; FGR: Fetal growth restriction; FM: Fetal movement; PCA: Principal component analysis.

## Competing interests

The authors declare that they have no competing interests.

## Authors’ contributions

BAW was responsible for data collection, quality assessment and classifications, statistical analyses, scientific interpretation of results, and writing of the manuscript. JR was responsible for the wavelet principal component analyses, statistical analysis, and for writing and revising the manuscript. ES, JE, CFR, and BSP contributed to data collection, scientific interpretations, and manuscript revision. JFF originally conceived of the study and contributed to scientific interpretation of the results and manuscript revision. All authors read and approved the final manuscript.

## Pre-publication history

The pre-publication history for this paper can be accessed here:

http://www.biomedcentral.com/1471-2393/13/172/prepub

## Supplementary Material

Additional file 1 Fetal movement chart. Click here for file
