# Supplementary material for: Wavelet principal component analysis of fetal movement counting data preceding hospital examinations due to decreased fetal movement: a prospective cohort study
Source: BMC Pregnancy Childbirth. 2013 Sep 5;13:172. doi: 10.1186/1471-2393-13-172 (PMC3844562; doi:10.1186/1471-2393-13-172)
Supplement: Additional file 1 — Fetal movement chart. [file 1471-2393-13-172-S1.pdf]

Kick count form, page 1

I started to count today (dd.mm.yy) ☐☐.☐☐.☐☐ Due date by ultrasound (dd.mm.yy) ☐☐.☐☐.☐☐ I usually count between (hours) ☐☐.☐☐ and (hours) ☐☐.☐☐

How to complete the kick count form

Use a blue or black pen.

For your convenience, fill in weekday and date in the appropriate box.

After counting 10 kicks/movements, record the number of minutes in the appropriate 5-minutes box on the kick count form.

Examples:

If it took 7 minutes, write 7 in the 6-10-minutes box.

If it took 1 hour and 5 minutes, write 65 in the 60-65 minutes box.

Week 24

	We	Th	Fr	Sa	Su	Mo	Tu
Date:	7/3	8/3					
Minutes:							
0-5							
6-10	7						
11-15							
16-20							
21-25							
26-30							
31-35							
36-40							
41-45							
46-50							
51-55							
56-60							
61-65		65					
66-70							
71-75							
76-80							
81-85							
86-90							
91-95							
96-100							
101-105							
106-110							
111-115							
116-120							
More than 2h? Record exact no of min.							

Fill in your study number if you are participating in *Count with me.*

	Week 24							Week 25							Week 26							Week 27							Week 28						
Weekday:																																			
Date:																																			
Minutes:																																			
0-5																																			
6-10																																			
11-15																																			
16-20																																			
21-25																																			
26-30																																			
31-35																																			
36-40																																			
41-45																																			
46-50																																			
51-55																																			
56-60																																			
61-65																																			
66-70																																			
71-75																																			
76-80																																			
81-85																																			
86-90																																			
91-95																																			
96-100																																			
101-105																																			
106-110																																			
111-115																																			
116-120																																			
More than 2h? Record exact no of min.																																			

Continue registration on page 2

Kick count form, page 2

	Week 29	Week 30	Week 31	Week 32	Week 33	Week 34	Week 35
Weekday:							
Date:							
Minutes:							
0-5							
6-10							
11-15							
16-20							
21-25							
26-30							
31-35							
36-40							
41-45							
46-50							
51-55							
56-60							
61-65							
66-70							
71-75							
76-80							
81-85							
86-90							
91-95							
96-100							
101-105							
106-110							
111-115							
116-120							
More than 2h? Record exact no of min.							

Continue registration on page 3

Kick count form, page 3

	Week 36	Week 37	Week 38	Week 39	Week 40	Week 41	Week 42
Weekday:							
Date:							
Minutes:							
0-5							
6-10							
11-15							
16-20							
21-25							
26-30							
31-35							
36-40							
41-45							
46-50							
51-55							
56-60							
61-65							
66-70							
71-75							
76-80							
81-85							
86-90							
91-95							
96-100							
101-105							
106-110							
111-115							
116-120							
More than 2h? Record exact no of min.							

REMEMBER TO RETURN YOUR KICK COUNT FORM AFTER DELIVERY. THANK YOU FOR YOUR PARTICIPATION.

HAVE YOU CONTACTED HEALTH CARE PERSONNEL DUE TO DECREASED FETAL ACTIVITY?

If so, please complete the questions below. Should you contact health care personnel on several occasions, please complete the questionnaire each time (use an extra sheet of paper if necessary).

1. Have you CONTACTED health care personnel because you have been worried about decreased activity? If so, when: ☐☐.☐☐.☐☐ (dd mm yy)

2. What did you DO?

I waited for ☐☐ hours before I CALLED:

- ☐ Antenatal care services/health clinic
- ☐ Maternity unit
- ☐ Other__________________________

I waited for ☐☐ hours before I VISITED

- ☐ Antenatal care services/health clinic
- ☐ Maternity unit
- ☐ Other__________________________

3. If you were EXAMINED, how did you perceive the baby's activity level at that time?

- ☐ The activity level was normal
- ☐ The activity level was still decrased

1. Have you CONTACTED health care personnel because you have been worried about decreased activity? If so, when: ☐☐.☐☐.☐☐ (dd mm yy)

2. What did you DO?

I waited for ☐☐ hours before I CALLED:

- ☐ Antenatal care services/health clinic
- ☐ Maternity unit
- ☐ Other__________________________

I waited for ☐☐ hours before I VISITED

- ☐ Antenatal care services/health clinic
- ☐ Maternity unit
- ☐ Other__________________________

3. If you were EXAMINED, how did you perceive the baby's activity level at that time?

- ☐ The activity level was normal
- ☐ The activity level was still decrased

1. Have you CONTACTED health care personnel because you have been worried about decreased activity? If so, when: ☐☐.☐☐.☐☐ (dd mm yy)

2. What did you DO?

I waited for ☐☐ hours before I CALLED:

- ☐ Antenatal care services/health clinic
- ☐ Maternity unit
- ☐ Other__________________________

I waited for ☐☐ hours before I VISITED

- ☐ Antenatal care services/health clinic
- ☐ Maternity unit
- ☐ Other__________________________

3. If you were EXAMINED, how did you perceive the baby's activity level at that time?

- ☐ The activity level was normal
- ☐ The activity level was still decrased

1. Have you CONTACTED health care personnel because you have been worried about decreased activity? If so, when: ☐☐.☐☐.☐☐ (dd mm yy)

2. What did you DO?

I waited for ☐☐ hours before I CALLED:

- ☐ Antenatal care services/health clinic
- ☐ Maternity unit
- ☐ Other__________________________

I waited for ☐☐ hours before I VISITED

- ☐ Antenatal care services/health clinic
- ☐ Maternity unit
- ☐ Other__________________________

3. If you were EXAMINED, how did you perceive the baby's activity level at that time?

- ☐ The activity level was normal
- ☐ The activity level was still decrased
